# Screening of ADHD symptoms in primary school students and investigation of parental awareness of ADHD and its influencing factors: A cross-sectional study

**DOI:** 10.3389/fpsyg.2022.1070848

**Published:** 2022-12-23

**Authors:** Hong-Hua Li, Tian-Tian Wang, Han-Yu Dong, Ya-Qin Liu, Fei-Yong Jia

**Affiliations:** ^1^Department of Developmental and Behavioral Pediatrics, The First Hospital of Jilin University, Changchun, Jilin, China; ^2^Pediatric Research Institute of Jilin, Changchun, Jilin, China

**Keywords:** attention deficit hyperactivity disorder, ADHD symptoms, screening rate, parents, awareness

## Abstract

**Objective:**

The present study aimed to compare the differences in positive screening rates of attention deficit hyperactivity disorder (ADHD) symptoms between parents and teachers in the same sample of primary school students. Concurrently, parental awareness and information sources of ADHD were investigated, and possible relevant factors affecting parental awareness and their influence on positive screening rate of ADHD were analyzed.

**Methods:**

A cross-sectional study was conducted in Changchun, China, between September 2020 and January 2021. Parents of 1,118 primary school students and 24 head teachers were recruited in the survey. Data were collected through a structured self-administered questionnaire. It consisted of socio-demographic characteristics, ADHD symptom screening questionnaire, parental awareness, and information sources of ADHD.

**Results:**

Among the 1,118 primary school students, 30 (2.7%) and 60 (5.4%) students were positive for Swanson, Nolan, and Pelham Rating Scale (SNAP-IV) screening in the parent version and teacher version, respectively. Parents had lower positive screening rates for ADHD symptoms than teachers. Relationship with children (mother, OR = 1.552, 95% CI = 1.104–2.180), bachelor degree or above of parents (OR = 1.526, 95% CI = 1.054–2.210), children’s sex (girl, OR = 1.442, 95% CI = 1.093–1.904), and age (OR = 1.344, 95% CI = 1.030–1.754), children’s grade (grade 2, OR = 0.522, 95% CI = 0.310–0.878; grade 3, OR = 0.388, 95% CI = 0.185–0.782), information sources of ADHD (medical staff, OR = 1.494, 95% CI = 1.108–2.015; family/relative/friend, OR = 1.547, 95% CI = 1.148–2.083; TV/Internet, OR = 3.200, 95% CI = 2.270–4.510) were the factors related to the parental awareness of ADHD.

**Conclusion:**

Parents and teachers of primary school students recognize ADHD symptoms differently. The positive screening rate of ADHD among teachers was significantly higher than that of parents. Relationship with children, educational level of parents, children’s sex, age, and grade, and information sources of ADHD are the relevant factors affecting parental awareness of ADHD. More efforts should be made to disseminate ADHD knowledge through mass media, and medical staff. Fathers, parents with low educational level, and parents of grade 2 and 3 pupils should be encouraged to acquire more knowledge on ADHD to improve the early recognition rate of ADHD symptoms.

**Clinical trial registration:**

[http://www.chictr.org.cn/showproj.aspx?proj=54 072], identifier [ChiCTR2000033388].

## 1 Introduction

Attention deficit hyperactivity disorder (ADHD) is a common neurodevelopmental disorder characterized by persistent and pervasive problems with inattention, hyperactivity, and impulsivity (Wolraich et al., 2019). ADHD usually occurs in early childhood before the age of 12 years (American Psychiatric Association, 2013). ADHD not only has an obvious impact on children and adolescence, such as poor interpersonal relationship, poor academic performance, low self-esteem, and negative emotions, but also makes patients more prone to anxiety, depression, and other mental health disorders (Klassen et al., 2004; Barkley, 2014; Nourredine et al., 2021). Moreover, for 40–60% of children with ADHD, the disorder will persist into adulthood, resulting in reduced mental health, and social wellbeing (Faraone et al., 2006; Agnew-Blais et al., 2018; Posner et al., 2020; Ren et al., 2021). Children with ADHD are more likely than their peers to develop conduct disorders and antisocial personality disorders in adulthood, consequently increasing the risk of substance use disorders and incarceration (American Psychiatric Association, 2013).

The global estimate of ADHD prevalence is 7.2% in children and 2.5% in adults (Thomas et al., 2015; Posner et al., 2020). A meta-analysis shows that the prevalence of ADHD among school-age children and adolescents in China is 6.3% (Liu et al., 2018). With such a high prevalence, perhaps only 10% of children with ADHD in China visit specialized clinics (Zheng and Liu, 2015). There are many possible reasons, such as the accessibility and convenience of ADHD clinic, the number of local pediatricians who have received professional training in ADHD, national culture and economic factors (Zheng and Zheng, 2015; Yu et al., 2019), among which the public’s lack of knowledge about ADHD and lack of awareness of the disease may also be one of the important reasons. Unrecognized and undiagnosed children may not receive standardized management and treatment, further reducing their quality of life.

The diagnosis and treatment of ADHD require close cooperation among doctors, teachers, and parents. The evaluation of children’s behavior by teachers and parents directly affects doctors’ judgment of children’s symptoms. Early identification of ADHD symptoms by parents and teachers can shorten the time from symptom onset to diagnosis of ADHD, contributing to early intervention and thus improving prognosis (Rocco et al., 2021). However, due to the different environments and perspectives of teachers and parents, their evaluation of children’s behavior may differ. Several studies have explored the consistency between parents and teachers in evaluating ADHD symptoms in the same group of children, and the results show a low consistency concerning ADHD symptoms and performance (Wolraich et al., 2004; Tripp et al., 2006; Holmberg et al., 2013). Teachers scored higher on inappropriate behavioral assessments, especially for externalized symptoms, such as hyperactivity and aggression (Tripp et al., 2006; Holmberg et al., 2013). Contrarily, Huang et al. (2017) used Conners’ 10-item scale to assess ADHD symptoms in school-age children and found that the positive screening rate of parents was significantly higher than that of teachers.

These inconsistencies may be related to the differences in the knowledge, understanding, and awareness of ADHD between parents and teachers. Particularly, parents’ insufficient awareness of ADHD makes it difficult for children to complete the entire assessment and treatment process at an early stage. Lack of awareness of ADHD leads to delay in recognizing ADHD and seeking appropriate treatment, which will lead to greater psychosocial problems (Park et al., 2018). Therefore, more importantly, parents should have good awareness and favorable attitudes toward ADHD. A retrospective review of 63 studies concluded that inadequate parental awareness of ADHD was one of the factors leading to poor treatment compliance (Corkum et al., 2015). However, in China, sporadic small sample studies indicate that parents may not have enough knowledge and awareness about ADHD (Huang et al., 2012).

In view of the problem and importance of early detection for effective treatment, this study aimed to assess and compare the differences in positive screening rates for ADHD symptoms between parents and teachers in the same sample of primary school students. Additionally, different from previous studies, parental awareness and information sources of ADHD were also investigated, and possible relevant factors affecting parental awareness and their influence on positive screening rate of ADHD were analyzed. Based on the differences in the assessment of ADHD symptoms by parents and teachers mentioned in the literature above, and the possibility that a lack of awareness of ADHD may lead to delayed diagnosis and treatment, we propose the following hypothesis: (A) Parents and teachers have different rates of positive screening for ADHD, and teachers have a higher rate of positive screening for ADHD. (B) Parental awareness of ADHD is influenced by education level and information sources of ADHD. (C) The higher the parental awareness of ADHD, the higher the positive screening rate of ADHD.

This study will provide evidence for early screening and diagnosis of ADHD, provide a reference for pediatricians to make a comprehensive clinical judgment and provide important details for future ADHD-related training programs.

## 2 Materials and methods

### 2.1 Study design

This study was a cross-sectional study and formed part of a nationwide survey of ADHD in primary schools of urban areas in China through the “Take Care of the Mental Health of the Youth” project. The study was conducted in two key and non-key primary schools in Changchun, China, between September 2020 and January 2021. The study protocol (2020-352) was reviewed and approved by the Research Ethics Committee of The First Hospital of Jilin University (Changchun, China). This study is registered in the Chinese Clinical Trial Registry (ChiCTR2000033388).

### 2.2 Sampling technique and participants

Firstly, Changchun is a provincial capital city with a population of more than 10 million, located in northeast China. It is also one of the 13 cities in the multi-center survey of primary school ADHD in urban areas of China (Chinese Clinical Trial Registry, 2022).

Secondly, a stratified two-stage cluster sampling technique was used. First, primary schools in the city zone provided by the Youth Digest Magazine were stratified by type of school into key and non-key schools. Thereafter, two key and non-key primary schools were selected through random sampling, keeping the principle of voluntary participations, which was to say, if the randomly selected schools were not willing to participate in the study, then the voluntary schools would be included automatically. In the random selection, we put all the alternative schools in order and assigned sequence labels. We used the computer to write a random number generation program math.random(). Each time we selected a sample, we used this program to randomly generate two numbers as a basis for selection. Subsequently, one class was randomly selected from each grade in each school, and 24 classes were selected in the study. Similarly, we sorted the classes for each grade and assigned sequence labels. Then we used the random number generation function to randomly generate one number each time to ensure the randomness of sample selection. The head teachers of the selected classes, and the parents of the classes eventually participated in the study.

The sample size of primary school students in this center was 1,118, which met the requirements of the national sample size calculation for each center: In the multi-center survey of ADHD primary school in urban areas in China, a sample size of 4,136 was required to detect ADHD with a positive rate of 7.2% and a 95% confidence interval (CI) of 1.6%. We assumed a 70% response rate. The sample size should be increased by half due to the group sampling method in this study. Therefore, 8,863 participants were required for the multi-center study. In order to increase the representativeness of the data, four schools were selected from each of the 13 cities. As a result, six classes were selected from each school, and a total of 312 classes were selected nationwide. In China, primary schools have at least 30 students in each class, so the final sample size is at least 9,360. The sample size of each center should be more than 720 cases.

The inclusion criteria included (1) acceptance and consent by parents and head teachers to participate in the study and (2) particularly for head teachers, a duration of at least 1 month as a head teacher for a class. If parents did not live with the child or were not proficient in Chinese, they were excluded from the study. Ultimately, 1,118 primary school students, their parents, and 24 head teachers were enrolled in the study.

### 2.3 Data collection tools

The data were collected using a structured self-administered questionnaire adapted from different literature. It consisted of socio-demographic characteristics, ADHD symptom screening questionnaire, parental awareness of ADHD, and information sources of ADHD.

#### 2.3.1 Socio-demographic data

The parents provided information on their relationship with children, age, time spent with the child each week, level of education, household per capita monthly income level, and child’s grade. The head teachers provided information on their age, sex, and years of teaching experience, title of their technical post, training on ADHD, and experience of teaching a child with ADHD.

#### 2.3.2 ADHD symptom screening questionnaire

The Swanson, Nolan, and Pelham Rating Scale (SNAP-IV) was used in this study to screen ADHD symptoms. SNAP-IV is an unstructured questionnaire on behavior completed by a child’s parent and teacher (Swanson et al., 2001). It consists of 26 items, including three dimensions of atypical behavior: inattention (nine items), hyperactivity/impulsivity (nine items), and oppositional defiant behaviors (eight items). Each item is scored according to the symptom severity, with a rating of 0 denoting “not at all,” 1 denoting “just a little,” 2 denoting “quite a bit,” and 3 denoting “very much.” The first two dimensions containing 18 items, inattention and hyperactivity/impulsivity, were used in this study. Both Chinese versions of SNAP-IV for teachers and parents had good reliability and validity (Gau et al., 2008; Gau et al., 2009).

Parents and head teachers completed the parent and teacher versions of the SNAP-IV rating scale, respectively, for each child. Thereafter, the positive screening rate of both versions of the SNAP-IV scale were calculated. A positive screening on the SNAP-IV Rating scale (Gau et al., 2008) was defined as at least six of nine items of the inattention dimension score ≥2 and/or at least six of nine items of the hyperactivity/impulsivity dimension score ≥ 2.

#### 2.3.3 Data on parental awareness of ADHD

This part of the questionnaire was used to assess the parental awareness of ADHD (consisting of 44 items about the symptoms, etiology, risk factors, treatment and medication knowledge, and perception of ADHD). These items were compiled based on a review of the literature (Bussing et al., 1998; McLeod et al., 2007; Bussing et al., 2012).

Each item was rated on a two-point (0 = no, 1 = yes) or was graded according to the degree of ADHD knowledge, with a rating of 0 denoting “not at all,” 1 denoting “just a little,” 2 denoting “quite a bit,” and 3 denoting “very much.” The total score was 47 points; a higher score indicated a better parental awareness of ADHD. The content reliability and validity of this part was approved by expert child and adolescent psychiatrists.

#### 2.3.4 Parents’ source of information on ADHD

These questions assessed potential sources of information on ADHD, such as medical staff, family member, relative, friend, school teachers, mass media programs about ADHD, internet, books or articles about ADHD, and leaflets about ADHD. Only two options were available as answers to these questions: yes and no.

### 2.4 Procedures

Two specific questionnaires were developed for this study: one for the parents of 1,118 primary school students enrolled in the study and the other for the 24 head teachers. A link to the questionnaire was sent to the parents through QR codes. Parents provided informed consent digitally, and completed the questionnaire. After obtaining the informed consent of parents, the 24 head teachers completed the teacher version of the SNAP-IV scale for each child in the class and questionnaire on their socio-demographic information. Finally, responses from the questionnaires were recovered through the electronic platform.

### 2.5 Statistical analyses

SPSS v22.0 (IBM, Armonk, NY, USA) was used for all analyses. The Kolmogorov–Smirnov test was used to evaluate the normality of the data distribution. Continuous data were presented as mean ± SD, median percentile (P50), P25, or P75, whereas categorical data were presented as frequencies with percentages. According to the distribution of the analyzed variable, an independent-sample *t*-test or a non-parametric Mann–Whitney *U*-test was used to compare continuous data of both groups. A chi-squared test was conducted to test the variation in the proportion of categorical variables among both groups. Parameters with *P* < 0.05 in univariable analysis between parents with good and poor awareness of ADHD and suspected correlation variables reported in previous literature were further assessed by multivariable logistic regression to analyze the correlation factors of parental awareness of ADHD. For the samples whose questionnaire content was obviously untrue or 90% of the questionnaire content was not completed, the data will be eliminated during the data analysis. *P* < 0.05 (two-sided) was considered statistically significant.

## 3 Results

### 3.1 Socio-demographic characteristics

Parents of all enrolled primary school students (*n* = 1,118) completed the parent version of SNAP-IV, and 24 head teachers also completed the teacher version of SNAP-IV. The socio-demographic characteristics of the parents and students participating in this study are shown in Table 1. Table 2 shows the demographic characteristics of the 24 head teachers participating in the study.

**TABLE 1:** Demographic characteristics of children and their parents (*n* = 1,118).

Variable	*n* (%), mean ± SD
**Relationship with children**	
Father	246 (22%)
Mother	850 (76%)
Grandparents	22 (2%)
Father’s age, years	38.6 ± 4.3
Mother’s age, years	36.8 ± 4.0
**Time spent with the child each week (hours)**	
<14	234 (21%)
14–36	279 (25%)
>36	605 (54%)
**Educational level of parents**	
Secondary or below	262 (24%)
Junior college	409 (36%)
Bachelor’s degree or above	447 (40%)
**Household per capita monthly income level (¥)**	
<5,000	485 (43%)
5,000–8,000	357 (32%)
>8,000	276 (25%)
**Child’s sex**	
Boy	602 (54%)
Girl	516 (46%)
The child’s age, years	8.5 ± 1.8
**Grade level**	
Grade 1	214 (19%)
Grade 2	226 (20%)
Grade 3	194 (17%)
Grade 4	207 (19%)
Grade 5	143 (13%)
Grade 6	134 (12%)
	

**TABLE 2:** Demographic characteristics of head teachers (*n* = 24).

Variable	*n* (%)
**Age, years**	
19–29	9 (37.5)
30–39	13 (54.2)
40–49	2 (8.3)
**Work experience, years**	
<5	13 (54.2)
5–10	4 (16.7)
11–19	5 (20.8)
20–29	2 (8.3)
**Sex**	
Women	24 (100)
Men	0 (0)
**Title of technical post**	
Undetermined	13 (54.2)
Primary	7 (29.2)
Intermediate	4 (16.7)
**Number of children with ADHD that you have taught in the past**		
0	10 (41.7)
1–4	12 (50)
5–9	2 (8.3)
**Number of previous ADHD-related training**	
Never received	20 (83.3)
1	4 (16.7)
	

### 3.2 Positive screening rates between parents and teachers for ADHD symptoms

Among the 1,118 primary school students, 30 (2.7%) students screened positive on the parent version of SNAP-IV, and 60 (5.4%) students screened positive on the teacher version of SNAP-IV. Parents had lower positive screening rates for ADHD symptoms than teachers (2.7% vs. 5.4%, χ^2^ = 10.419, *P* = 0.001).

Among them, 53 (4.7%) students were screened negative by parents but positive by teachers. Twenty (2.1%) students were screened negative by teachers but positive by their parents; seven (0.6%) students were screened positive by both parents and teachers. The total number of parents and teachers who provided positive screening was 76, and the total positive screening rate was 6.8%.

### 3.3 Differences between parents and teachers in SNAP-IV screening for ADHD symptoms in the primary school students

No significant differences were found in the average age, sex, and grade composition between the students in the positive parent screening group and the positive teacher screening group (Table 3). The SNAP-IV scores on both versions were compared. In the inattention dimension of SNAP-IV, no significant difference was present in the total score, average score of each item, and the number of positive items between both groups. However, in the hyperactivity/impulsivity dimension, the total score, average score of each item, and number of positive items assessed by teachers were significantly higher than those assessed by parents (Table 3).

**TABLE 3:** Comparison of demographic and attention deficit hyperactivity disorder (ADHD) symptoms between children with positive parental screening and positive teacher screening.

Variable	Positive screening of the parents (*n* = 30)	Positive screening of the teachers (*n* = 60)	χ ^2^/*t*/*Z*	*P*
**Sex**				
Boy	20 (67%)	43 (72%)	0.238	0.626
Girl	10 (33%)	17 (28%)		
Age (mean ± SD)	8.7 ± 1.7	9.1 ± 1.8	−0.988	0.326
Grade Level			−1.152	0.249
Grade 1	5 (17%)	8 (13%)		
Grade 2	4 (13%)	4 (7%)		
Grade 3	5 (17%)	10 (17%)		
Grade 4	10 (33%)	21 (35%)		
Grade 5	4 (13%)	8 (13%)		
Grade 6	2 (7%)	9 (15%)		
Total score (ADHD-IA) P50 (P25, P75)	19 (17, 22)	20 (16, 26)	−0.374	0.708
The average score for each item (ADHD-IA) P50 (P25, P75)	2.1 (1.9, 2.4)	2.2 (1.8, 2.8)	−0.374	0.708
Number of positive items (ADHD-IA) P50 (P25, P75)	7 (6, 9)	8 (6, 9)	−1.263	0.207
Total score (ADHD-HI) P50 (P25, P75)	13 (7, 17)	17 (11, 24)*	−2.09	0.037
The average score for each item (ADHD-HI) P50 (P25, P75)	1.4 (0.8, 1.9)	1.9 (1.2, 2.6)*	−2.09	0.037
Number of positive items (ADHD-HI) P50 (P25, P75)	4 (1, 7)	7 (2, 9)*	−1.997	0.046
Positive only in ADHD-IA, *n* (%)	19 (63%)	25 (42%)	3.757	0.053
Positive only in ADHD-HI, *n* (%)	2 (7%)	6 (10%)	Fisher	0.714
Positive in both ADHD-IA and ADHD-HI, *n* (%)	9 (30%)	29 (48%)	2.756	0.097

ADHD-IA, inattention dimension in SNAP-IV; ADHD-HI, hyperactivity/impulsivity dimension in SNAP-IV.

*P < 0.05 compared with the positive screening of the parents’ group.

The positive screening rate in the inattention dimension was higher in the parent screening group than that in the teacher screening group (63% vs. 42%, *P* = 0.053). The positive screening rate in the inattention and hyperactivity/impulsivity dimensions in the parent screening group was lower than that in the teacher screening group (30% vs. 48%, *P* = 0.097). This finding indicated that teachers had a higher ability to identify hyperactivity and impulsivity symptoms, although the differences were not statistically significant (Table 3).

### 3.4 Comparison of the demographic and ADHD information sources of parents with different levels of ADHD awareness

The parents of all 1,118 primary school students participated in the survey of ADHD awareness and information sources of ADHD, but 185 of them were excluded due to incomplete responses to the questionnaires. With the median score of the ADHD awareness questionnaire as cut-off point (*n* = 933, median score: 27), 933 parents were divided into good awareness group (total score ≥27) and poor awareness group (total score <27). Parents’ socio-demographic data and ADHD information source of the two groups are listed in Table 4. The total positive screening rate of the two groups for ADHD was 23/933 (2.5%). The positive screening rate for ADHD was higher in the good awareness group than that in the poor awareness group (3.0% vs. 1.8%, *P* = 0.233), although the difference was not statistically significant.

**TABLE 4:** Comparison of the demographic and attention deficit hyperactivity disorder (ADHD) information sources between parents with good and poor awareness of ADHD (*n* = 933).

Variable	Poor awareness group (*n* = 439)	Good awareness group (*n* = 494)	χ^2^/*Z*	*P*
Total score of ADHD awareness questionnaire P50 (P25, P75)	23 (21, 25)	30 (28, 33)*	−26.439	<0.001
Positive screening rate for ADHD *n* (%)	8 (1.8%)	15 (3.0%)	1.425	0.233
Relationship with children			6.460	0.04
Father	112 (55%)	92 (45%)		
Mother	317 (45%)	390 (55%)*		
Grandparents	10 (45.5%)	12 (54.5%)		
Father’s age, years	38 (35, 41)	39 (36, 41)	−1.863	0.062
Mother’s age, years	36 (34, 39)	37 (35, 40)*	−2.722	0.006
Time spent with the child each week (hours)			2.754	0.252
<14	88 (46%)	103 (54%)		
14–36	121 (52%)	113 (48%)		
>36	230 (45%)	278 (54%)		
Educational level of parents			4.140	0.126
Secondary or below	122 (53%)	109 (47%)		
Junior college	155 (46%)	185 (54%)		
Bachelor’s degree or above	162 (45%)	200 (55%)		
Household per capita monthly income level (¥)			1.663	0.435
<5,000	194 (47%)	215 (53%)		
5,000–8,000	147 (49%)	152 (51%)		
>8,000	98 (44%)	127 (56%)		
Child’s sex			3.901	0.048
Boy	247 (50%)	246 (50%)		
Girl	192 (44%)	248 (56%)*		
The child’s age, years	8 (7, 10)	9 (7, 10)*	−2.266	0.023
Grade level			13.025	0.022
Grade 1	77 (46%)	92 (54%)		
Grade 2	98 (54%)	82 (46%)		
Grade 3	80 (55%)	65 (45%)		
Grade 4	65 (40%)	98 (60%)*		
Grade 5	63 (44%)	79 (56%)*		
Grade 6	56 (42%)	78 (58%)*		
**Source of information about ADHD**				
Medical staff	160 (42%)	222 (58%)*	6.934	0.008
Family/Relatives/Friends	159 (42%)	224 (59%)*	7.999	0.005
TV/Internet	285 (41%)	419 (60%)*	49.689	<0.001
Printed publications (Newspapers/Periodicals/Books/Handbooks)	96 (37%)	164 (63%)*	14.845	<0.001
Teachers	168 (48%)	181 (52%)	0.263	0.608
A patient or parent with ADHD	23 (26%)	65 (74%)*	17.063	<0.001

*P < 0.05 compared with the poor awareness group.

No significant differences in father’s average age, weekly time spent with the child, educational level of parents, and household per capita monthly income level existed between the two groups. Compared with the poor awareness group, the good awareness group had a higher participation rate among mothers than among fathers, higher average ages of mothers and children, higher proportion of girls, and higher proportion of children enrolled in grades 4, 5, and 6 (Table 4).

The proportions of ADHD-related knowledge obtained from medical staff, family/relatives/friends, TV or internet, paper publications, and patients or parents with ADHD in the good awareness group were significantly higher than those of the poor awareness group (Table 4).

### 3.5 Factors associated with parental awareness level of ADHD

Relationship with children (father or mother), educational level of parents at the bachelor’s degree or higher level, sex and age of the child, children’s grade and ADHD information obtained from medical staff, family/relatives/friends, and TV or internet were the relevant factors that were associated with parental awareness level of ADHD (Table 5).

**TABLE 5:** Results of multivariate logistic regression concerning factors associated with parental awareness level of attention deficit hyperactivity disorder (ADHD).

Variable	*B*	S.E.	*P*	OR (95% CI)
Mother’s age, years *n* (%)	0.015	0.019	0.437	1.015 (0.978–1.053)
Relationship with children			0.031*	
Mother	0.439	0.174	0.011*	1.552 (1.104–2.180)
Educational level of parents			0.079	
Bachelor’s degree or above	0.422	0.189	0.025*	1.526 (1.054–2.210)
**Child’s sex**				
Girl	0.366	0.142	0.01*	1.442 (1.093–1.904)
Child’s age, years	0.296	0.136	0.029*	1.344 (1.030–1.754)
Grade Level			0.047*	
Grade 2	−0.65		0.014*	0.522 (0.310–0.878)
Grade 3	−0.97		0.009*	0.388 (0.185–0.782)
Grade 4	−0.61		0.194	0.542 (0.215–1.365)
Grade 5	−1.12		0.061	0.328 (0.102–1.053)
Grade 6	−1.32		0.068	0.266 (0.064–1.101)
**Source of information about ADHD**				
Medical staff	0.401	0.153	0.008*	1.494 (1.108–2.015)
Family/Relatives/Friends	0.436	0.152	0.004*	1.547 (1.148–2.083)
TV/Internet	1.163	0.175	<0.001*	3.200 (2.270–4.510)
Printed publications	0.217	0.170	0.203	1.242 (0.89–1.733)
A patient or parent with ADHD	0.431	0.296	0.145	1.539 (0.861–2.748)

Outcome variable = with good awareness of ADHD, 1 = yes, 0 = no. B, regression coefficient; S.E., standard error; OR, odds ratio; CI, Confidence interval. *P < 0.05.

## 4 Discussion

This study elicited three main findings. First, a significant difference existed between teachers and parents in the positive screening rate of ADHD symptoms in primary school students, and the positive screening rate of teachers was significantly higher than that of the parents, this is consistent with our first hypothesis. Second, teachers scored higher on hyperactivity and impulsivity symptoms than parents, and the recognition rate of these symptoms showed an upward trend. Third, relationship with children, educational level of parents, children’s sex, age, grade, and parental information sources about ADHD were the factors related to the parental awareness of ADHD.

Children are often unable to reliably report their own behaviors. An ADHD diagnosis requires not only an examination of the child but also interviews with parents and data from teachers about the child’s behavior at school (de Nijs et al., 2004). Therefore, accurate recognition of ADHD symptoms by parents and teachers is necessary for diagnosis. However, this study shows that a significant difference exists in the recognition of ADHD symptoms by parents and teachers in primary school students. Only 0.6% of children were screened positive by both parents and teachers, which is far lower than the rate reported by international data on ADHD epidemiology (Thomas et al., 2015). Some studies (Wolraich et al., 2004; Tripp et al., 2006; Murray et al., 2007; Holmberg et al., 2013) have applied questionnaires assessing strengths and difficulties and Conners’ scale to evaluate the consistency of parents’ and teachers’ scoring of ADHD symptoms; the results showed low consistency between responses by parents and teachers. Narad et al. (2015) revealed that parent and teacher ratings of ADHD behaviors are only weakly-to-moderately correlated. The low agreement between parents and teachers may be related to the situational specificity of ADHD symptoms at home and at school (Gomez, 2007). Attention deficit, hyperactivity, and impulsive symptoms in children are more likely to be observed in a school setting where teachers encounter students, especially regarding the latter symptoms. Murray et al. (2007) found that children at risk of ADHD exhibit significant differences in behavior patterns across settings. In addition, parents and teachers assume different roles and may have different expectations of children’s behavior (DuPaul, 2020). As teachers, they may pay more attention to children’s ability to sustain attention, not to interfere with class activities, and be seated for a long time. Whereas parents may prioritize their children’s abilities, such as doing homework, doing housework, and the ability to have a good relationship with peers. Last but not least, the low agreement may be related to the differences in awareness or perception of ADHD symptoms between parents and teachers (Huang et al., 2012; Park et al., 2018). However, in this study, only 24 head teachers participated in the screening of ADHD symptoms. Due to the obvious differences in the sample size between teachers and parents, we did not conduct a comparative analysis on their awareness of ADHD. In future studies, we will further sample and investigate the differences in awareness of ADHD between primary school teachers and parents.

Knowledge of ADHD has a positive correlation with perception of ADHD (Hosseinnia et al., 2020). Therefore, the higher the awareness of ADHD is, the more likely ADHD symptoms will be detected early. This study investigated parental awareness of ADHD, and possible relevant factors affecting their awareness were analyzed, as well as their influence on positive screening rate of ADHD. The results showed that nearly 50% of parents scored below 60% of the total score on the ADHD awareness questionnaire, suggesting that parents’ overall awareness of ADHD was low. These findings are consistent with those of McLeod et al. (2007) and Speerforck et al. (2021), which found a low level of public knowledge and beliefs about ADHD. Due to parents’ low knowledge and awareness of ADHD, their recognition ability is insufficient, which leads to parents’ inability to consult a specialist clinic in time. This may also be the reason for the low positive screening rate for ADHD symptoms among parents (2.3%), which is much lower than the global ADHD prevalence rate of 7.2% (Thomas et al., 2015). In this study, the positive screening rate for ADHD was higher in the good awareness group than that in the poor awareness group (3.0% vs. 1.8%), although the difference was not statistically significant. This finding suggests that parents with good awareness of ADHD tend to increase the recognition rate of ADHD, which also confirms our hypothesis.

In addition, consistent with McLeod et al. (2007) and Gerdes et al. (2021), this study found that mothers’ awareness of ADHD was significantly higher than fathers’. Women are more likely to recognize and acknowledge the existence of psychiatric problems, and are more predisposed than men to seek treatment for health problems (Horwitz, 1977; Drapalski et al., 2009). Conversely, fathers of children with ADHD have strong resistance to biomedical explanations and medication strategies for ADHD. They tend to be largely absent from ADHD-related research and clinical settings, which may lead to their lower awareness of ADHD (Singh, 2003). Additionally, this study found that parents with higher education had higher awareness of ADHD, which is also consistent with our second hypothesis. The similarity has been reported by others (McLeod et al., 2007). Dodangi et al. (2017) reported that parents’ knowledge of ADHD was significantly correlated with their educational level. Most likely, a higher educational level afforded parents the increased capacity to acquire knowledge on childhood-related health issues, including ADHD.

Interestingly, parents of girls were more aware of ADHD than parents of boys, and no study has reported similar findings. This finding seems to be contrary to conventional understanding. Compared with boys with ADHD, girls with ADHD are less likely to be referred for clinical assessments due to a lower prevalence of externalizing symptoms (Chai et al., 2021; Hinshaw et al., 2021; Chronis-Tuscano, 2022). However, a retrospective observational study by De Rossi et al. (2022) showed that ADHD features in girls were more severe than those in boys in clinically referred settings. Therefore, we speculate that parental awareness of ADHD might be related to the severity of children’s ADHD symptoms, which needs to be further explored. In addition, this study showed that children’s age was a relevant factor affecting parents’ awareness of ADHD, and parents of older children had a higher awareness of ADHD. This may be related to the characteristics of ADHD symptoms. With increasing age of children with ADHD, functional impairments become more pronounced due to the persistence of ADHD symptoms (Agnew-Blais et al., 2018; Ren et al., 2021). Therefore, parents are more likely to be aware of ADHD. Interestingly, parents of grade 2 and 3 pupils had lower awareness of ADHD than parents of grade 1 pupils. This finding may be related to the fact that parents pay more attention to children’s adaptability in the collective environment, and communicate more closely with teachers when children enter the first year of primary school. Therefore, they were more alert to find behavioral disorders.

Additionally, this study revealed that the higher the proportion of ADHD information obtained from medical staff, family or friends, TV or internet, the higher the awareness of ADHD. This finding reveals that these are the main sources for parents to acquire knowledge on ADHD. Comparable results were reported by Dodangi et al. (2017), Alshehri et al. (2020), and Dessie et al. (2021). This suggests that the publicity and popularization of basic knowledge on ADHD should be increased through mass media, and medical staff should increase the popularization of ADHD knowledge to improve parental awareness of ADHD. Comprehensive training programs offered by pediatricians can improve parents’ understanding of ADHD and adherence to medication (Zheng et al., 2020; Shen et al., 2021). Therefore, parents, especially fathers, parents with lower level of education, and parents of grade 2 and 3 pupils should be encouraged to acquire more knowledge on ADHD, which might help improve public awareness of ADHD and early recognition rate of ADHD.

This study had some limitations. First, we only compared the positive screening rate of ADHD between parents and teachers, and did not make further diagnosis of ADHD for children with positive screening. Second, since only few head teachers participated in the study, the influence of the differences in awareness of ADHD between parents and teachers on the positive screening rate was not compared. Third, although the content reliability of the parent ADHD awareness questionnaire was endorsed by child and adolescent psychiatrists, its reliability was not verified in our sample. Fourth, sampling was only performed in the urban area of Changchun, and no investigation was conducted on ADHD of rural children, which limited the generalizability of the current findings. Future studies should expand the study population to include children in rural areas. Fifth, this study was a cross-sectional survey; therefore, causal relations could not be established. Likewise, if subsequent training had been provided, another survey could have been conducted to assess the change in knowledge on ADHD. Therefore, future psychoeducational programs on ADHD with much more advanced approaches are still needed.

## 5 Conclusion

Recognition of ADHD symptoms among parents and teachers of primary school students differed. The positive screening rate of ADHD among teachers was significantly higher than that of parents. Teachers have higher rates of recognition of hyperactivity and impulsivity symptoms. Relationship with children, educational level of parents, children’s sex, age, and grade, and information sources of ADHD were the relevant factors affecting parental awareness of ADHD. More effort should be made to disseminate ADHD knowledge through mass media, and medical staff. Fathers, parents with low educational level, and parents of primary school students in grade 2 and 3 should be encouraged to actively acquire knowledge on ADHD to hopefully improve the early recognition rate of ADHD symptoms.

## Data availability statement

The original contributions presented in this study are included in this article/supplementary material, further inquiries can be directed to the corresponding author.

## Ethics statement

The studies involving human participants were reviewed and approved by the Research Ethics Committee of The First Hospital of Jilin University. Written informed consent to participate in this study was provided by the participants or their legal guardian/next of kin.

## Author contributions

F-YJ and H-HL designed the work. H-HL collected and analyzed the data, and drafted the manuscript. T-TW, H-YD, and Y-QL collected the data and revised the manuscript. F-YJ contributed to the concept of this study and revised the manuscript. All authors contributed to the article and approved the submitted version.
